# Case Report: Dupilumab as a corticosteroid-sparing adjunct in severe mucocutaneous pemphigus vulgaris with prior avascular necrosis and chronic kidney disease

**DOI:** 10.3389/fmed.2026.1859006

**Published:** 2026-06-26

**Authors:** Qingbi Hu, Yalan Yan, FengCong Liu, Chunshui Yu, Linli Liu

**Affiliations:** 1 Department of Dermatology, Sichuan Provincial People's Hospital East Sichuan Hospital, Dazhou First People's Hospital, Dazhou, China; 2 North Sichuan Medical College, Nanchong, China; 3 Chengdu University of Traditional Chinese Medicine, Chengdu, China; 4 Department of Dermatology, Suining Central Hospital, Suining, China

**Keywords:** autoimmune blistering disease, avascular necrosis, chronic kidney disease, corticosteroid-sparing therapy, dupilumab, pemphigus vulgaris

## Abstract

Pemphigus vulgaris is a potentially life-threatening autoimmune blistering disease for which corticosteroid minimization can be difficult when prior steroid toxicity has already occurred. We report a 60-year-old woman with severe mucocutaneous pemphigus vulgaris confirmed by clinical, immunopathologic, and serologic findings, who had previously developed femoral head avascular necrosis during high-dose prednisone therapy and also had advanced chronic kidney disease, hypoalbuminemia, and anemia. Rituximab was discussed as a guideline-supported first-line option; however, after counseling regarding its expected benefits and potential adverse effects, the patient declined rituximab. Therefore, dupilumab was introduced off-label as an adjunctive corticosteroid-minimizing approach together with oral prednisone 30 mg/day and supportive care. The Pemphigus Disease Area Index was 111 during the first treatment week and decreased to 22 by week 6, when disease control was achieved with cessation of new lesions and marked healing of pre-existing erosions. Prednisone was then tapered gradually. Complete re-epithelialization was achieved by approximately month 4 while the patient was receiving oral prednisone 7.5 mg/day, and clinical stability was maintained through month 6 after dupilumab discontinuation at month 4. No adverse events were observed. This case supports the clinical feasibility of dupilumab-assisted corticosteroid minimization in selected patients with pemphigus vulgaris and major treatment-related constraints, while further evidence is needed regarding patient selection, duration of therapy, and long-term relapse risk.

## Introduction

Pemphigus vulgaris (PV) is a rare, potentially life-threatening autoimmune blistering disease characterized by flaccid blisters and painful erosions involving the skin and mucous membranes (1). Standard treatment relies on systemic corticosteroids combined with steroid-sparing immunosuppressants and/or rituximab (1). However, management remains challenging in patients with prior glucocorticoid intolerance or glucocorticoid-related complications, in whom conventional corticosteroid exposure may be undesirable.

Dupilumab is a fully human monoclonal antibody targeting interleukin-4 receptor alpha (IL-4Rα), thereby inhibiting IL-4 and IL-13 signaling (2). Emerging case-based evidence suggests that dupilumab may have therapeutic potential in pemphigus, although the available data remain limited and heterogeneous (3–5). Here, we report a case of severe mucocutaneous PV in a patient with prior steroid-related toxicity, including avascular necrosis of the femoral head. Rituximab was considered and discussed as a guideline-supported first-line option, but the patient declined rituximab after counseling because of concerns about potential adverse effects. Dupilumab was therefore introduced off-label as an adjunctive corticosteroid-minimizing approach in combination with a reduced-dose oral prednisone regimen. This case is presented to describe clinical decision-making in a constrained real-world setting, not to suggest that dupilumab should replace guideline-supported rituximab therapy.

## Case description

### Diagnostic assessment

A 60-year-old woman presented to the Department of Dermatology with recurrent generalized erythema, flaccid blisters, and painful erosions. According to the patient, her symptoms initially began more than 1 year prior without an identifiable trigger. At that time, she was diagnosed with pemphigus vulgaris at another institution and initiated on high-dose oral corticosteroids (prednisone 60 mg/day). Although the blistering initially responded to the treatment, she subsequently developed severe glucocorticoid-related complications, most notably avascular necrosis of the femoral head. This debilitating adverse event necessitated a premature and rapid tapering of the corticosteroids, which unfortunately precipitated a severe disease relapse. Over the preceding month, her mucocutaneous lesions had significantly aggravated despite topical supportive care. Her complex medical history was further complicated by concomitant advanced chronic kidney disease (CKD), raising substantial therapeutic challenges for selecting a conventional steroid-sparing agent. Her family history was noncontributory. She reported no known family history of pemphigus, autoimmune blistering disease, systemic autoimmune disease, atopic disease, chronic kidney disease, or malignancy among first-degree relatives.

**Figure 1:**
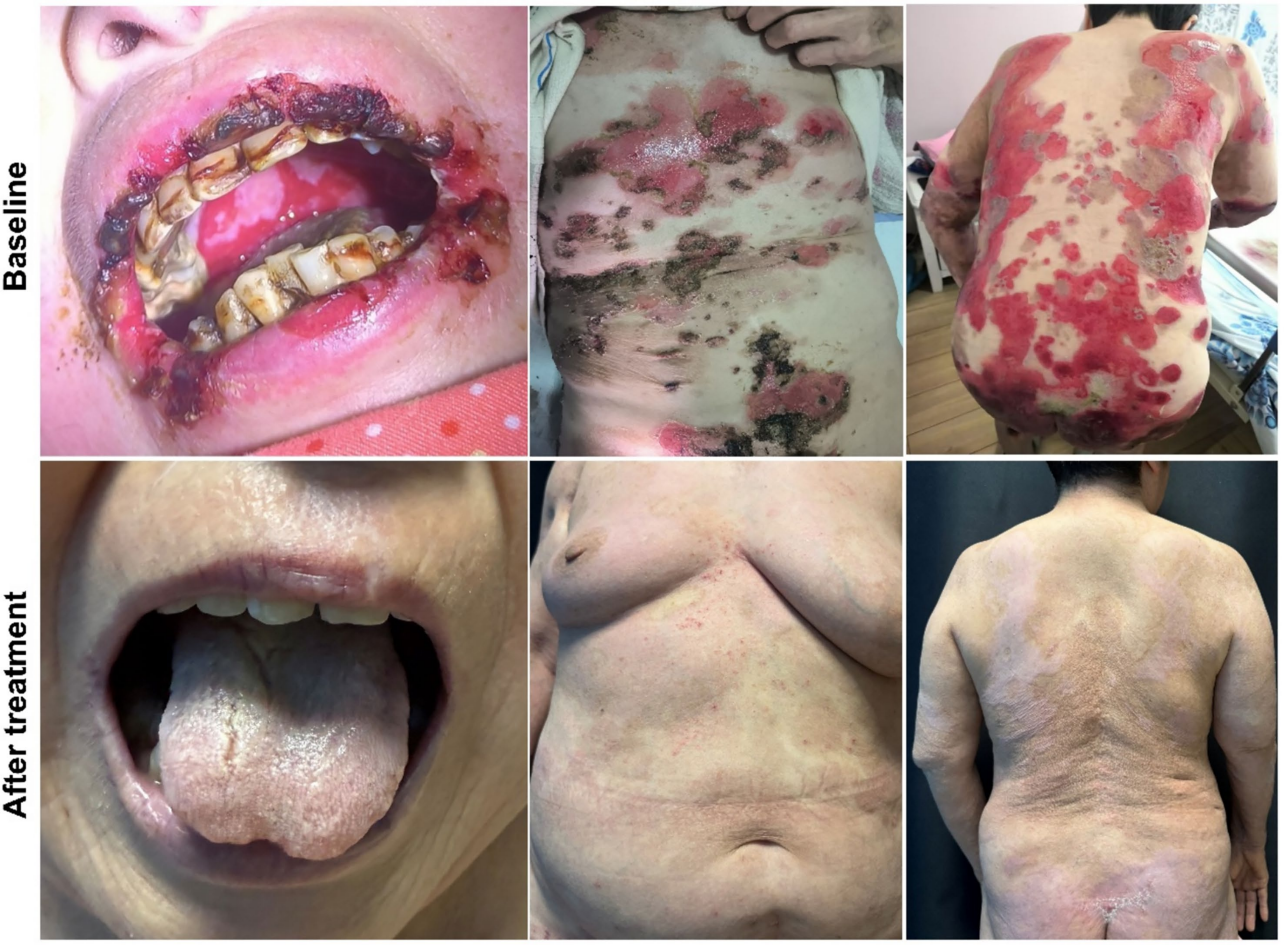
Clinical manifestations of pemphigus vulgaris before and after dupilumab-based therapy. **(A–C)** Baseline: extensive mucocutaneous involvement with painful erosions and hemorrhagic crusting of the lips and oral mucosa **(A)**, widespread erythematous erosions/crusted plaques on the trunk **(B)**, and confluent erosions and erythematous plaques on the back **(C)**. **(D–F)** Images obtained at the 6-month follow-up visit, approximately 2 months after dupilumab discontinuation, showing marked re-epithelialization of the oral mucosa with resolution of crusting **(D)** and near-complete clearance of cutaneous erosions on the trunk and back with residual post-inflammatory pigmentary changes **(E–F)**.

**Figure 2:**
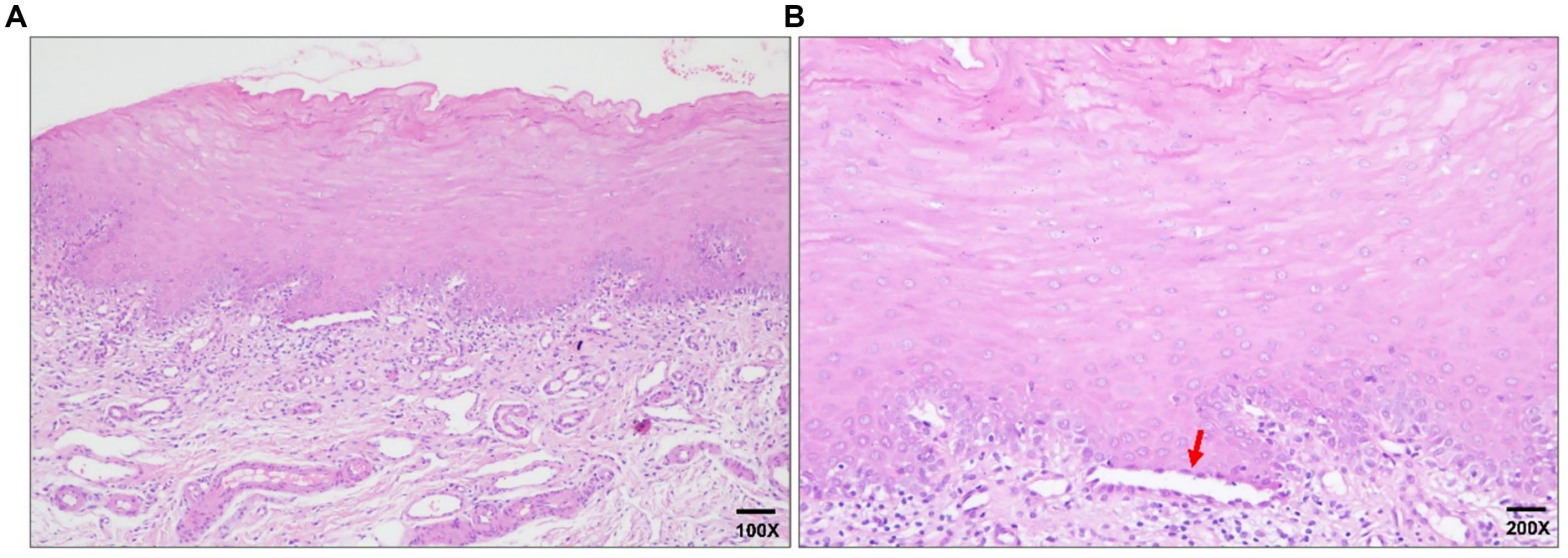
Histopathological findings of the mucosal biopsy. **(A)** Low-power view shows chronic mucosal inflammation with intraepithelial cleft/blister formation. **(B)** High-power view demonstrates an intraepithelial blister/cleft (arrow) with inflammatory cell infiltration in the underlying lamina propria. Hematoxylin and eosin (H&E) stain; original magnifications ×100 **(A)** and ×200 **(B)**.

**Figure 3:**
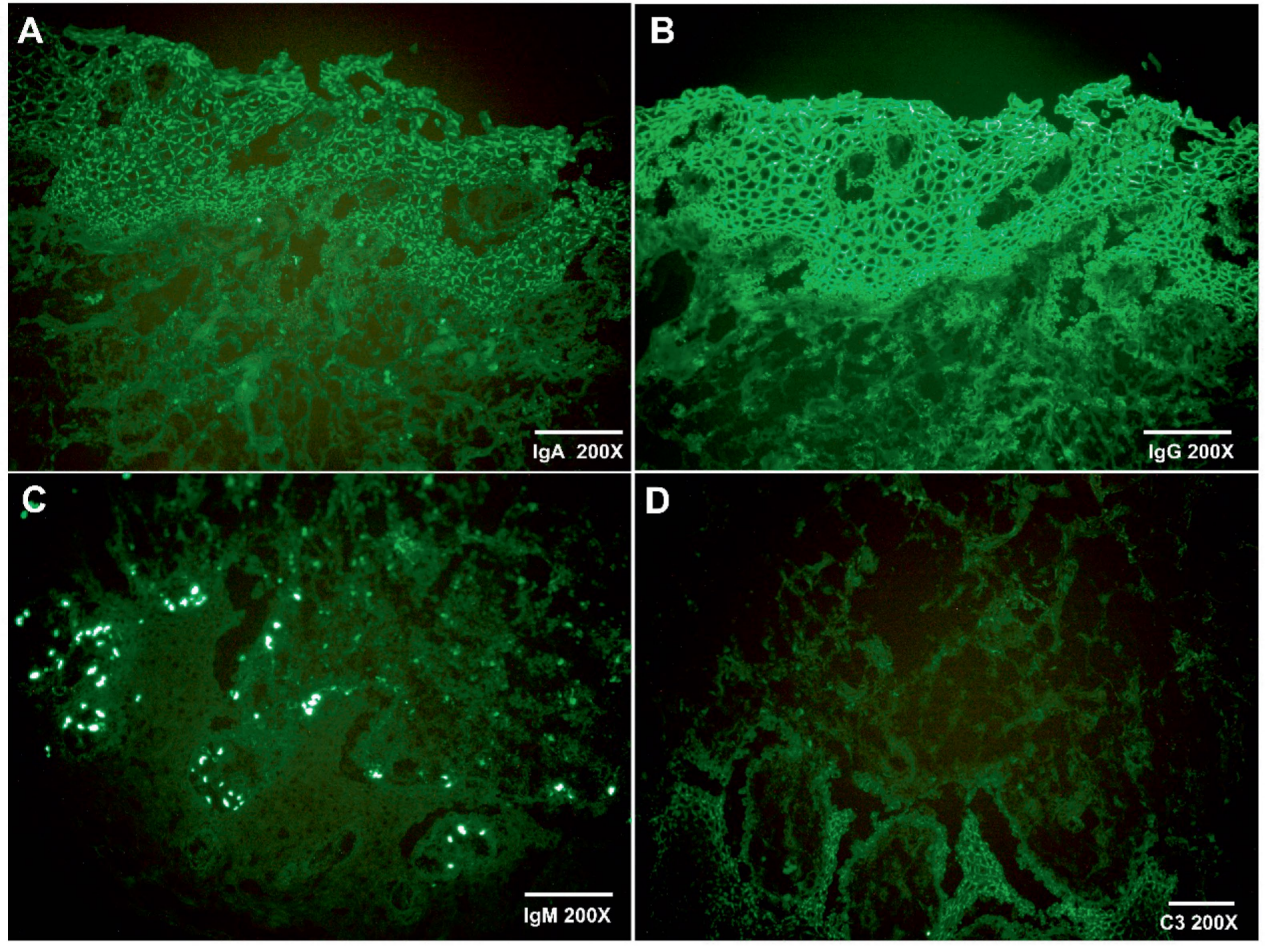
Direct immunofluorescence (DIF) findings of perilesional mucosa. Intercellular (“fishnet-like”) fluorescence is observed in the stratified squamous epithelium for IgA **(A)** and IgG **(B)**, with C3 positivity **(D)** in the intercellular spaces. IgM is negative **(C)**. Original magnification ×200.

On physical examination, erosions with exudation and hemorrhagic crusting were observed at the canthi, lips, and oral cavity. The chest, back, and extremities showed densely distributed erythematous patches and erosions of variable size, partially confluent, with exudation and hemorrhagic crusts. Nikolsky’s sign was positive (Figures 1A–C). Histopathological examination of an oral mucosal biopsy showed chronic mucosal inflammation with focal intraepithelial cleft/blister formation, suggestive but not definitive for pemphigus (Figures 2A,B). Direct immunofluorescence performed on perilesional oral mucosa demonstrated intercellular (“fishnet-like”) deposition of IgG (++), with IgA (+) and C3 (+), whereas IgM was negative (Figures 3A–D). Serological testing revealed positivity for anti-desmoglein 3 (Dsg3) antibodies, while anti-Dsg1, anti-BP180, and anti-BP230 antibodies were negative. Laboratory tests showed a red blood cell count of 2.63 × 10^12/L, hemoglobin 78 g/L, albumin 24 g/L, creatinine 543.8 μmol/L, urine protein (++), and urine occult blood (+++). Based on the clinical presentation, immunopathological findings, and serology, a diagnosis of mucocutaneous pemphigus vulgaris (PV) was established, complicated by hypoalbuminemia, moderate anemia, and advanced chronic kidney disease. The episode of care is summarized in Table 1.

**Table 1:** Timeline of the episode of care.

Time point	Clinical course and key findings	Intervention/Outcome
>1 year before current admission	Recurrent blisters and erosions began without an identifiable trigger. The patient was diagnosed with pemphigus vulgaris at another institution.	Oral prednisone 60 mg/day was initiated, with initial clinical improvement.
Subsequent prior course	Steroid-related avascular necrosis of the femoral head developed during prior high-dose corticosteroid therapy. Rapid tapering was required.	Severe disease relapse followed premature corticosteroid reduction.
1 month before admission	Marked aggravation of mucocutaneous lesions despite topical supportive care.	The patient was admitted for further evaluation and treatment.
Day 0 (admission)	Generalized erythema, flaccid blisters, painful oral erosions, and positive Nikolsky sign were documented. Direct immunofluorescence showed intercellular IgG (++), with IgA (+) and C3 (+). Anti-Dsg3 antibodies were positive, whereas anti-Dsg1, anti-BP180, and anti-BP230 antibodies were negative. Hemoglobin was 78 g/L, albumin 24 g/L, and creatinine 543.8 μmol/L.	Subcutaneous dupilumab 600 mg loading dose was administered with oral prednisone 30 mg/day. Topical treatment included recombinant bovine basic fibroblast growth factor gel and fusidic acid cream applied to cutaneous lesions, mouth rinses with 3% sodium bicarbonate solution for oral mucosal care, intravenous albumin supplementation, and nutritional support.
Week 1	Baseline disease severity was documented.	PDAI 111 (skin 46; mucosa 65).
Week 2	Early treatment phase.	Dupilumab 300 mg every 2 weeks was started. Oral prednisone 30 mg/day was initially maintained.
Week 6	No new lesions developed and pre-existing erosions had markedly healed.	Disease control was achieved. PDAI decreased to 22 (skin 19; mucosa 3). Oral prednisone taper was initiated.
Month 4	Complete re-epithelialization was achieved while the patient was receiving low-dose prednisone.	Oral prednisone had been tapered to 7.5 mg/day. Dupilumab was discontinued.
Month 6	Clinical stability was maintained without relapse or reported adverse events.	The patient remained on oral prednisone 7.5 mg/day.

### Therapeutic intervention

Because minimizing cumulative corticosteroid exposure was a treatment priority in view of the patient’s prior steroid-related toxicity, particularly femoral head avascular necrosis, a corticosteroid-minimizing regimen was prioritized in the initial inpatient treatment plan. Rituximab was considered and discussed with the patient as a guideline-supported first-line treatment option for moderate-to-severe PV. The potential benefits, mode of administration, possible adverse effects, infection-related risks, and treatment logistics of rituximab were explained. After counseling, the patient expressed substantial concern about the potential adverse effects of rituximab and declined rituximab at that time. Under these real-world treatment constraints, dupilumab was selected off-label through shared decision-making as an adjunctive corticosteroid-minimizing approach rather than as a guideline-preferred replacement for rituximab. After informed consent, the patient received a 600 mg subcutaneous loading dose of dupilumab on day 0, combined with oral prednisone 30 mg/day. Topical and symptomatic treatment was also provided. Cutaneous erosions and crusted lesions were managed with topical recombinant bovine basic fibroblast growth factor gel to promote epithelial repair and topical fusidic acid cream when secondary bacterial infection was suspected or prevention of local infection was clinically indicated. Oral mucosal care consisted of mouth rinses with 3% sodium bicarbonate solution. Supportive treatment included albumin supplementation and nutritional support. From week 2 onward, subcutaneous dupilumab 300 mg was administered every 2 weeks while the prednisone dose was initially maintained.

### Follow-up and outcomes

The Pemphigus Disease Area Index (PDAI) documented during the first treatment week was 111 (skin 46; mucosa 65). The patient’s body weight at treatment initiation was 60 kg; therefore, the initial oral prednisone dose of 30 mg/day corresponded to 0.5 mg/kg/day. This dose was selected to avoid further high-dose corticosteroid exposure in view of her prior steroid-related avascular necrosis. By week 6, disease control was achieved, defined by the absence of new lesions and marked healing of pre-existing erosions, and the PDAI had decreased to 22 (skin 19; mucosa 3), indicating substantial overall improvement with a particularly marked mucosal response. Oral prednisone was then tapered by 5 mg every 2 weeks. Once the dose reached 20 mg/day, tapering was individualized to 2.5–5 mg every 2–4 weeks according to disease activity. Complete re-epithelialization was achieved after approximately 4 months of treatment while the patient was receiving oral prednisone 7.5 mg/day (Figures 1D–F). Dupilumab was discontinued after 4 months, and the patient was maintained on oral prednisone 7.5 mg/day. At 6-month follow-up, she remained clinically stable on oral prednisone 7.5 mg/day without relapse, and no adverse events were reported.

## Discussion

This case illustrates the challenges of managing severe mucocutaneous PV when further high-dose corticosteroid exposure is undesirable and a guideline-supported first-line option is not pursued in a specific real-world context. Rituximab remains an evidence-supported first-line therapy for moderate-to-severe PV, and neither chronic kidney disease nor prior corticosteroid-related avascular necrosis is an absolute contraindication to rituximab. In the present case, rituximab was discussed with the patient, but she declined rituximab because of concerns about potential adverse effects after counseling. Therefore, dupilumab should not be interpreted as a replacement for rituximab. Rather, it was used off-label as an adjunctive corticosteroid-minimizing strategy after individualized shared decision-making.

To date, evidence supporting dupilumab in pemphigus remains limited to case reports and small case series, with heterogeneous regimens and outcomes (3–5). In the case reported by Chen et al., dupilumab was given for 10 weeks as add-on therapy with moderate-dose systemic glucocorticosteroids and intravenous immunoglobulin; clinical improvement was observed, but complete systemic corticosteroid withdrawal was not reported, as low-dose methylprednisolone plus dupilumab was continued after discharge (3). Moore and Hurley described short-term dupilumab monotherapy for recalcitrant PV after prior corticosteroid therapy, with dose escalation to weekly injections because oral lesions recurred before the next scheduled dose (4). In the series by Jiang et al., one PV patient maintained remission through 63 weeks of dupilumab treatment with extension of the dosing interval to every 6 weeks, whereas another developed new oral and cutaneous lesions 4 weeks after dupilumab initiation during concomitant prednisone tapering and discontinued treatment (5). Overall, the available PV reports suggest a potential corticosteroid-sparing role for dupilumab, but complete prednisone-free remission has not been consistently documented. Dupilumab was generally well tolerated where reported, with no adverse events noted in the Jiang et al. series, although clinical responses were variable (5).

PV has evidence-based management recommendations, and steroid-sparing strategies are emphasized to reduce cumulative corticosteroid toxicity (1). In the present case, the rationale for introducing dupilumab was not primarily renal dysfunction, but rather the need to reduce glucocorticoid burden because of prior steroid-related toxicity, particularly femoral head avascular necrosis. Prednisone was gradually tapered to 7.5 mg/day, which is consistent with commonly used definitions of minimal therapy when maintained without new or established lesions (6). Given the severe baseline disease activity, the marked improvement by week 6 and maintenance on oral prednisone 7.5 mg/day at 6 months appear clinically notable and may suggest a potential adjunctive corticosteroid-sparing contribution of dupilumab, although causal attribution remains limited by the concomitant use of oral prednisone, topical therapy, and supportive care.

Advanced CKD did not absolutely preclude dupilumab use, but it complicated therapeutic selection. Several conventional steroid-sparing agents can be more difficult to use in advanced renal impairment because nephrotoxicity, renal clearance, or exposure changes may complicate dosing and monitoring. In contrast, dupilumab is a fully human monoclonal antibody handled primarily through catabolic pathways rather than clinically meaningful renal elimination (7). Therefore, while the primary rationale for initiating dupilumab was to minimize further corticosteroid-related toxicity, the presence of advanced chronic kidney disease further supported consideration of dupilumab as a corticosteroid-sparing option in this patient.

Beyond the established role of pathogenic autoantibodies, accumulating data suggest that a type 2-skewed immune milieu may contribute to autoimmune blistering diseases, including pemphigus and bullous pemphigoid (BP) (8). Dupilumab has also shown clinical activity in BP (9, 10), providing additional biologic plausibility for IL-4Rα blockade in selected PV patients. The IL-4/IL-13 axis is central to type 2 inflammation and can promote humoral immune responses (2). Accordingly, targeting IL-4/IL-13 signaling has been proposed as a potentially actionable strategy in pemphigus (2, 8).

This report has several limitations. First, it describes a single patient and includes concomitant systemic corticosteroids, precluding causal attribution of efficacy to dupilumab alone. Second, serial objective measures such as repeat PDAI scoring and/or Dsg3 titers were not available to quantify response over time. Third, dupilumab was discontinued at month 4, and follow-up after discontinuation remains limited; therefore, the long-term relapse risk and the optimal duration of dupilumab therapy in PV remain unknown. Finally, supportive measures, including topical therapy and nutritional support, may also have contributed to clinical improvement. Finally, rituximab was not used in this patient because she declined it after counseling regarding its benefits and potential risks; therefore, this report should not be interpreted as evidence that dupilumab is comparable or superior to guideline-supported rituximab therapy. Nevertheless, this case describes the clinical feasibility of an off-label dupilumab-based corticosteroid-minimizing approach in a selected PV patient under specific real-world treatment constraints.

### Patient perspective

Before this admission, the lesions on my skin and in my mouth kept recurring and gradually became severe enough to affect my daily life. I was very worried about receiving high-dose corticosteroids again because my previous treatment had caused serious problems with my hip. After the new treatment was started, the erosions gradually improved and I felt more comfortable, especially when eating and speaking. I was also relieved that the steroid dose could be reduced step by step. During follow-up, I did not notice any obvious side effects from dupilumab. I understand that longer follow-up is still needed, but I am grateful that my condition became stable.

## Data Availability

The original contributions presented in the study are included in the article/supplementary material, further inquiries can be directed to the corresponding authors.
